# Bell’s measure and implementing quantum Fourier transform with orbital angular momentum of classical light

**DOI:** 10.1038/srep14113

**Published:** 2015-09-15

**Authors:** Xinbing Song, Yifan Sun, Pengyun Li, Hongwei Qin, Xiangdong Zhang

**Affiliations:** 1 School of Physics, Beijing Institute of Technology and Beijing Key Laboratory of Fractional Signals and Systems, 100081, Beijing, China; 2 Department of Physics, Beijing Normal University, Beijing 100875, China

## Abstract

We perform Bell’s measurement for the non-separable correlation between polarization and orbital angular momentum from the same classical vortex beam. The violation of Bell’s inequality for such a non-separable classical correlation has been demonstrated experimentally. Based on the classical vortex beam and non-quantum entanglement between the polarization and the orbital angular momentum, the Hadamard gates and conditional phase gates have been designed. Furthermore, a quantum Fourier transform has been implemented experimentally.

Bell’s measure is commonly used in tests of quantum non-locality; it has attracted much attention in the last years due to the possibility of ruling out classical hidden-variable theories^1^,^2^,^3^,^4^,^5^. Recently, it has been demonstrated that Bell’s measure can also be used as a quantitative tool in classical optical coherence^6^. Non-separable correlations among two or more different degrees of freedom from the same classical optical beam have been discussed^6^,^7^,^8^,^9^,^10^,^11^,^12^,^13^,^14^,^15^,^16^,^17^,^18^,^19^. The violation of Bell’s inequality for such a non-separable correlation has been demonstrated experimentally^6^,^7^,^8^,^9^. Such a non-separable classical correlation is called “non-quantum entanglement” or “classical entanglement”^10^,^11^,^12^,^13^,^14^,^15^,^16^,^17^,^18^,^19^. It has been applied to resolve basic issues in polarization optics^10^, simulate quantum walks, etc^12^.

So far, the classical entanglement between polarizations and some spatial modes such as spatial parity and Hermite modes, has been demonstrated experimentally in polarized beams of light^6^,^7^,^8^. On the other hand, vortex beams with various orbital angular momenta (OAM) have been experimentally realized in the optical domain^20^,^21^,^22^,^23^. The possibility of encoding large amounts of information in vortex beams due to the absence of an upper limit has raised the prospects of their applicability in quantum information processing tasks, such as computation and cryptography^22^,^23^. Although Bell-like inequality for the spin-orbit separability of a laser beam has been discussed^7^, direct Bell’s measurement for the non-separable correlation between polarizations and OAM from the same classical vortex beam has not been done.

In this work, we perform direct Bell’s measurement between polarization and OAM from the same classical vortex beam, and explore classical entangled properties between them. Because the vortex beam can carry OAM with any mode number, such properties are expected to have more extensive application. Based on such a non-quantum entanglement, we implement the quantum Fourier transform (QFT), which is the crucial final step in Shor’s algorithm^24^,^25^,^26^,^27^,^28^,^29^,^30^. Comparing it with the quantum realization, we find that the classical implementation of QFT exhibits many advantages. High calculation efficiency that the QFT possesses is not only retained, the experiment can also be designed in a relatively simple way. We hope that our study could be an important reference for both classical and quantum information processing.

## Results and Discussion

### Bell’s measurement for the correlation between polarization and orbital angular momentum

**Figure 1:**
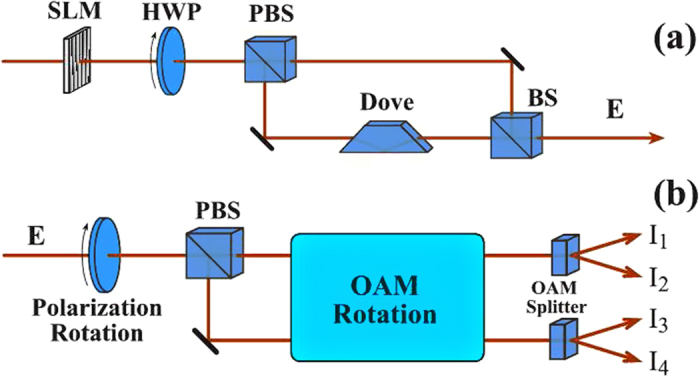
(**a**) The preparation of classical entangled states for polarization and orbital angular momentum. (**b**) Experimental setup for CHSH-type Bell’s measurement for the correlation between polarization and orbital angular momentum. SLM is spatial light modulator, HWP is half-wave plate, PBS is polarizing beam splitter, BS is 50/50 beam splitter, Dove is Dove prism, and OAM represents orbital angular momentum.

We consider a light beam being manipulated with a spatial light modulator (SLM) passing through a half-wave plate (HWP) and a polarizing beam splitter (PBS) as shown in Fig. 1(a). After the PBS, the light beam is divided into two intensity equaled parts for the two paths with horizontal 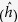 and vertical 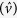 polarizations, respectively. In the path with vertical polarization, a Dove prism is introduced. Then, the two polarized vortex beams in two paths are combined by a beam splitter (BS), and the output of the vortex beam can be expressed as


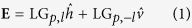


where LG_*p,l*_ represents the *l*th Laguerre function and *p* is the radial mode number. If the horizontal and vertical polarization components of the vortex beam are described by a slightly modified version of the familiar bra-ket notation of quantum mechanics, 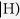 and 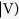
14, the OAM of *LG*_*p,l*_ are expressed by 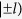, the polarized vortex beam can be described by the ket notation


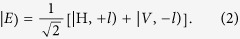


This representation for the polarized vortex beam is formally equivalent (isomorphic) to a Bell state of two polarized qubits. Hence, the polarization and OAM may be treated as two qubits that are classically entangled. Such an entanglement (local entanglement) can be realized from a single light beam. The problem is whether or not such an entanglement relation can be demonstrated by Bell’s measurements.

In order to answer such a problem, an experiment was designed as shown in Fig. 1(b). A polarized vortex beam passes through a HWP and a PBS, and splits into two beams. Then they are manipulated with OAM rotation systems and splitter devices to become four beams. The output intensities of four vortex beams are marked in Fig. 1(b) by *I*_1_, *I*_2_, *I*_3_ and *I*_4_, respectively. In order to perform the Clauser–Horne–Shimony–Holt (CHSH) Bell’s measurement, we define the following correlation function^6^:


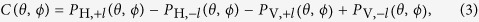


where *θ* and *ϕ* represent polarization and OAM rotated angles in the paths, respectively. The 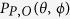 (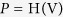 for the polarization and 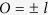 for the OAM number) are normalized probabilities of states on the certain measurement basis, which can be obtained through the intensity measurements in the experiments, that is 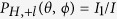, 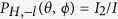, 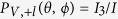, 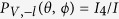 and *I* = *I*_1_ + *I*_2_ + *I*_3_ + *I*_4_. After we have obtained *C*(*θ*, *ϕ*), the CHSH measurement is


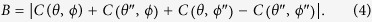


**Figure 2:**
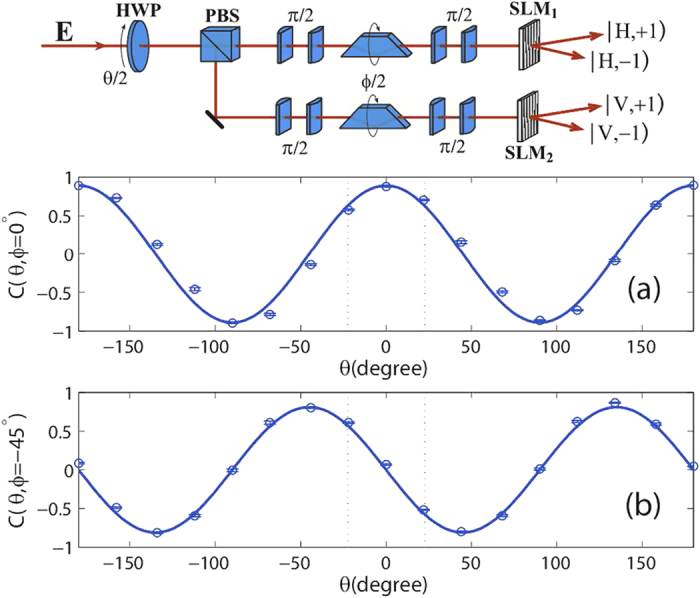
The correlation functions *C*(*θ*, *ϕ*) as a function of polarization rotation angle *θ* at (**a**) *ϕ* = 0° and (b) *ϕ* = −45°. The round dots and solid lines represent the experimental and theoretical results, respectively. The dashed lines mark the values of *θ* required to achieve the maximum violations of Bell inequalities. Experimental setup for the CHSH-type Bell’s measurement for the correlation between polarization and orbital angular momentum is shown at the top of the figure.

Figure 2(a,b) show experimental results for the correlation function *C*(*θ*, *ϕ*) from the same vortex beam with 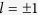 as a function of *θ* and *ϕ*. In the experiment, the vortex beam is produced by the diffraction from a phase hologram pattern on the SLM as described in ref. 21. Here the OAM rotation has been realized by using Dove prisms and conventional *π*/2 astigmatic mode converters^31^, and the OAM splitter has been implemented by the SLMs as shown in the experimental setup at the top of Fig. 2. It is well known that the polarization changes when the incident beam passes through the rotated Dove prism^32^,^33^. In our experiments, some certain values of Dove prism’s rotation angle are taken. Thus, the effect of such a phenomenon on the experimental results is very small.

A continuous laser with wavelength 632.8 nm is used. The round dots and solid lines represent the experimental measurements and the theoretical results, respectively. Here, the theoretical results are normalized by the experimental data, such that the amplitude maximum of the theoretical curve are taken to be equal to the maximum of the experimental data. It can be seen that the experimental results are in good agreement with the theoretical calculations in its changing character. If we take 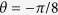, 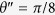, 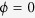 and 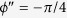, 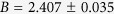 is obtained. Although there is some imprecision in the measurements and imperfections in the optical elements, the presented experimental results yield a violation of Bell’s inequalities.

**Figure 3:**
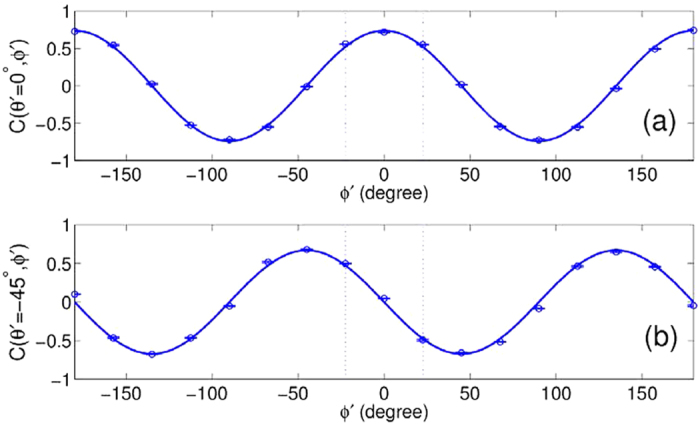
The correlation functions 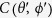 as a function of polarization rotation angle 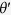 at (a) 0° and (b) −45° for *l* = 2. The round dots and solid lines represent the experimental and theoretical results, respectively. Here 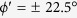, 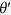 and *ϕ*′ still represent polarization and OAM rotation angles, but their values are different from *θ* and *ϕ* in Fig. 2 (see Methods).

The experimental results in Fig. 2 only exhibit the classical correlation between the polarization and the OAM with 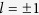. In principle, the classical correlation between the polarization and the OAM with any mode number can also be tested through such an experimental design. However, it is very difficult to operate in this scheme. Thus, we take another scheme, for which the detailed design has been given in the Methods section. The experimental results for 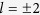 are plotted as round dots in Fig. 3. The solid lines are theoretical results, which are normalized by the experimental data. An agreement between the experimental measurements and theoretical results in changing character is observed again. If we take 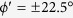, 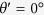 and 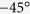, 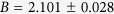 is obtained. Comparing the results in Fig. 3 with those for 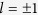 in Fig. 2, we find that the experimental results still yield a violation of Bell’s inequalities, although the loss in the experimental process increases for the present case with 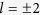. With the increase of *l*, it becomes more difficult to obtain the measured results with B > 2 because it requires a more accurate measurement of the intensity and precise operation of optical elements. However, the violation of Bell’s inequalities can be confirmed.

This means that the presence of local classical entanglement between the polarization and the OAM has been demonstrated. The question is whether or not such classical entanglement can be exploited to perform algorithms for quantum information processing. Because the QFT is the crucial final step in some quantum algorithms such as Shor’s algorithm, in the following we explore the possibility to realize QFT by using the local classical entanglement between the polarization and the OAM.

### Quantum Fourier transform based on the classical vortex beam

The QFT is a basis transformation in an N-state space that transforms the state 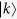 according to


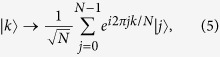


where 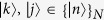 and 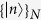 is a set of complete orthogonal basis vectors with *N* dimensions, *k* and *j* represent an integer ranging from 0 to *N* − 1. So far, the QFT has been demonstrated experimentally by using a quantum Hadamard gate and conditional phase gates^34^,^35^. The main advantage of QFT against the classical Fourier transformation is that of higher calculation efficiency, which originates from the quantum correlations.

The above investigations have shown that classically entangled states can exhibit similar correlation properties with the quantum correlations. If we take the classical states 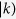 and 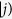 instead of the quantum states 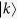 and 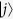, a similar transformation 
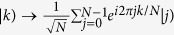
 can be achieved by using the local classical entanglement between the polarization and the OAM. In the following, we take two-qubit as an example to demonstrate such a process. The polarization degree of freedom is marked as the first qubit, that is 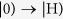 and 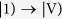, the OAM is marked as the second qubit, 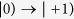 and 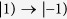, then the four Bell’s states that we use as an example to show our QFT experiment, are expressed as:


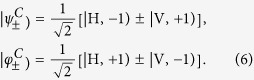


**Figure 4:**
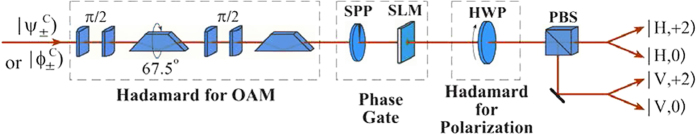
Experimental setup for the quantum Fourier transform (QFT) by using the local classical entanglement state between the polarization and orbital angular momentum. The QFT is composed of two Hadamard transforms and one conditional phase gate. The SPP represents the spiral phase plate.

In order to realize the QFT of the four Bell states, we present an experimental setup as shown in Fig. 4. It consists of two Hadamard gates and one controlled phase gate. The first Hadamard gate is for the OAM, which has been realized by using two Dove prisms and two mode converters. The rotation of OAM by two mode converters (*π*/2) and a Dove prism with horizontal angle 67.5° can be expressed as a matrix: 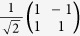. The function of the second Dove prism with horizontal angle 0° is to realize 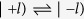. Combining the second Dove prism, the rotation matrix of OAM becomes 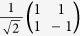. In this way, the Hadamard transformation for the OAM is realized. The second Hadamard gate is for the polarization, which can be realized by a HWP.

The controlled phase gate consists of one spiral phase plate (SPP) (RPC photonics, VPP-m633) and one SLM. The function of the SPP is to change the order of OAM. Here we use it to realize 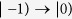 and 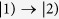. Thus, the two light beams with OAM 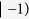 and 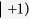, which can not be separated in the space, can be transformed to spatially separable modes 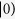 and 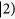 in the space. In such a case, we can do operation on different modes at the same time with an optical element whose function differs in the space. For example, a*π*/2 phase can be added only to 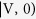 (OAM 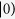 and vertical polarization) by using the SLM. Let the four Bell states pass through such an experimental setup; the output states are:


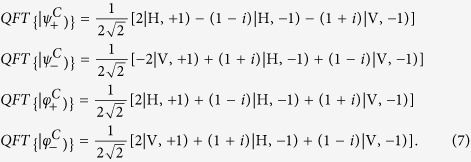


In order to obtain the information from the output states, we measure the intensity by using a PBS and a diaphragm as shown in Fig. 4. That is to say, measure the output intensities for four basis vectors 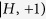, 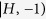, 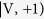 and 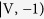. After the controlled phase gate, four basis vectors become 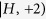, 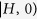, 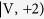 and 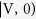, respectively. Then they pass through a PBS and the field intensities with different OAM can be obtained by blocking the light in space. They should correspond to the modular squares of coefficients for the four basis vectors, if the QFT has been realized.

**Figure 5:**
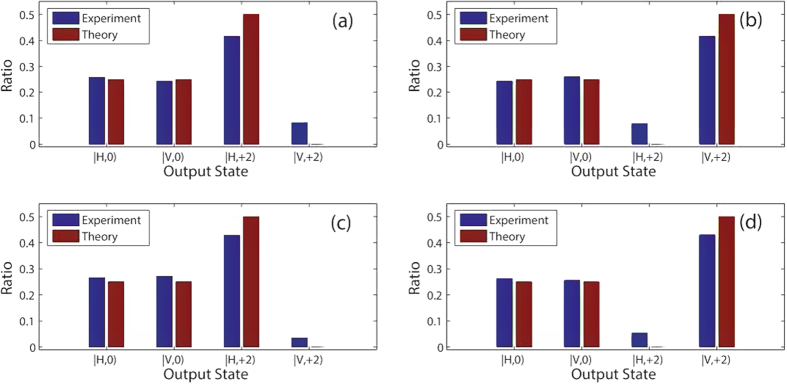
Comparison between experimental results and theoretical calculations for the QFT. (**a**–**d**) exhibit output ratios between the measured intensities for the four output states (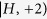, 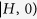, 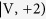 and 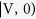 corresponding to 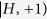, 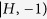, 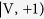 and 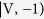), and the total intensity for the four input states (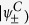 and 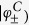), respectively. The blue bars represent theoretical results, and the red bars are experimental results. (**a**) corresponds to 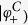; (**b**) to 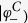; (**c**) to 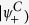; (d) to 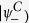.

In Fig. 5, we present the comparison between the theoretical computation and the experimental measurements. Figure 5(a–d) show output ratios of different basis vectors for the four kinds of input states, respectively. Here the output ratio represents the ratio of measured intensity for each basis vector and the total output intensity. The blue bars represent theoretical results, and the red bars are experimental results. Comparing them, we find that the agreements between the theoretical results and experimental measurements are in good agreement. This means that the QFT has been realized by our experimental setup. We would like to point out that the above results are only for the classical vortex beam with *l* = ±1, and the QFT can be realized by using a classical vortex beam with any mode number if ideal optical elements and methods are achieved.

## Conclusions

In summary, Bell’s measurement for the non-separable correlation between the polarization and the OAM from the same classical vortex beam has been performed experimentally. The violations of Bell’s inequalities for the non-separable classical correlations with various OAM have been demonstrated experimentally. Based on the non-quantum entanglement between the polarization and the OAM in the classical vortex beam, the Hadamard gates and controlled phase gates have been designed, and the QFT has been implemented experimentally. Such an implementation of QFT exhibits many advantages compared with the usual quantum realizations. For example, it is not only easier to implement, the measurement efficiency is also high. Moreover, it sheds light on the new concept of non-quantum entanglement. In the present work, a basic idea has been presented to realize two-qubit QFT via classical beams. In fact, if we can extend such a method to multi-qubit QFT, it could be surely used in a primary case of Shor’s algorithm. The expansion of the present work to simulate quantum computing, or perform other quantum information processing task, is a subject for future research.

## Methods

**Figure 6:**
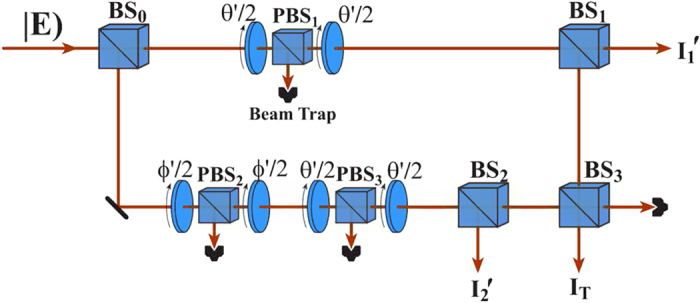
Experimental setup for the CHSH-type Bell’s measurement of the correlation between polarization and OAM with any mode number.

In order to perform Bell’s measurement for the correlation between the polarization and the OAM with 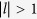 more efficient, we consider the experimental setup as shown in Fig. 6, which is similar to that in Ref. 36. In contrast to the scheme in Fig. 2, in the present scheme the measurement basis for the OAM has been generated by controlling the polarization and the projection measurement can be realized by using the interference method. Then, the output intensities 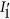, 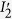 and *I*_T_ as marked in Fig. 6 can be obtained. The calculated processes for 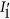, 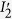 and *I*_T_ are given in the following:

The Jones matrix for the optical element group consisting of two HWPs and one PBS can be expressed as 
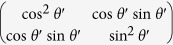
, where the fast axis direction for the HWP is taken as 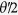 and the transmission light from the PBS is in the horizontal polarization. For convenience, the input state can be expressed as: 
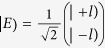
, where the first line and the second line of the column matrix correspond to 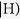 and 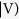, respectively. From Fig. 6, the 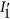 can be calculated in the following process:


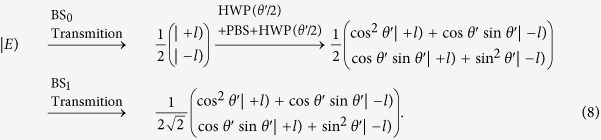


Considering the orthogonality of OAM modes, 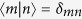, we can obtain


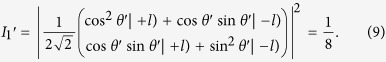


For the 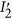, we have


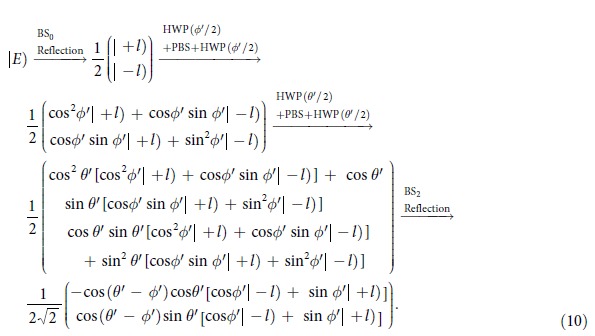


Here a *π* phase has been added in the horizontal polarized mode and the reversal has been happened for the OAM modes by the reflection, that is 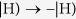 and 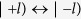. Then


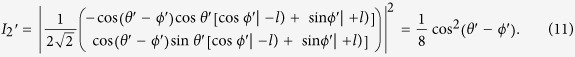


The *I*_T_ represents the output field intensity from the BS_3_, and the input field comes from the reflection from the BS_1_ and the transmission from the BS_2_. The reflection field from the BS_1_ is expressed as: 
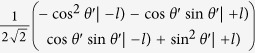
, and the transmission field from the BS_2_ is expressed as: 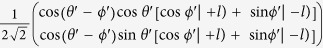. We consider the Jones matrix 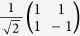 and the influence of reflection on the polarization and OAM, the output field from the BS_3_ can be expressed as:


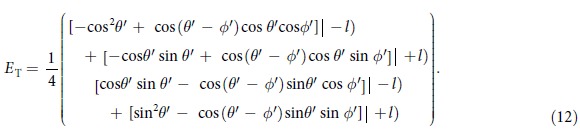


Then, 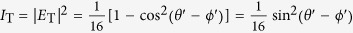.

After 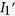, 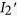 and *I*_T_ have been obtained, the normalized probabilities for the polarization and the OAM states 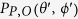 can be expressed as 
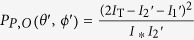
,^36^ here *I* represents the input total intensity, 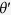 and *ϕ*′ still represent polarization and OAM rotated angles. Compared with the expression in the above section, we use 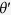 and 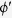 instead of *θ* and *ϕ* because they come from different elements in the experiments. Then, 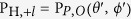, 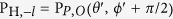, 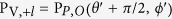 and 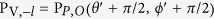. The correlation function 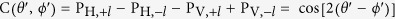 and the CHSH measurement B can also be obtained. The calculated results are shown in Fig. 3.

## Additional Information

**How to cite this article**: Song, X. *et al.* Bell's measure and implementing quantum Fourier transform with orbital angular momentum of classical light. *Sci. Rep.* **5**, 14113; doi: 10.1038/srep14113 (2015).
